# The impact of State of Surrender on the relationship between engagement in substance use treatment and meaning in life presence: a pilot study

**DOI:** 10.3389/fpsyg.2024.1331756

**Published:** 2024-06-17

**Authors:** Thomas B. Sease, Cathy R. Cox, Amanda L. Wiese, Emily K. Sandoz, Kevin Knight

**Affiliations:** ^1^Institute of Behavioral Research, College of Science and Engineering, Texas Christian University, Fort Worth, TX, United States; ^2^Department of Psychology, Texas Christian University, Fort Worth, TX, United States; ^3^Department of Psychology, University of Louisiana at Lafayette, Lafayette, LA, United States

**Keywords:** state of surrender, treatment engagement, peer support, counselor rapport, meaning in life

## Abstract

The current study examined the construct of State of Surrender (SoS)—defined as a willingness to accept, without resistance, what is to come—and investigated SoS as a statistical mediator of the relationship between engagement in substance use treatment and meaning in life (MIL). Using a cross-sectional design, participants were 123 people involved with the legal system participating in a 6-month residential treatment program for substance use. Results showed that measures of treatment engagement, including treatment participation, counselor rapport, and peer support, were all positively associated with SoS scores (*R*^2^s ≥ 21.16). Moreover, while controlling for time spent in treatment, SoS statistically mediated the positive association between aspects of treatment engagement and MIL. State of Surrender may be a targetable process in substance use treatment that aids in recovery by orienting clients toward what they find meaningful in life. Future directions and practical considerations are discussed.

## 1 Introduction

The Federal Bureau of Prisons (2023) reported that 45% of people incarcerated in the United States are serving time for a drug-related offense. Moreover, it has been estimated that more than half of the people involved with the legal system meet the diagnostic criteria for a substance use disorder (SUD; Bronson et al., 2017). Troubles with substance use have been correlated with diminished physical and psychological well-being (Keaney et al., 2011; Lai et al., 2015; McKetin et al., 2019; Colledge et al., 2020), a heightened risk of returning to custody post-release (Zgoba et al., 2020), and increased all-cause mortality rates (Hakansson and Berglund, 2013; Hayes, 2013; Chang et al., 2015). People in the legal system with a SUD are also more likely to return to substance use following a period of incarceration (Chamberlain and Boggess, 2019). For this reason, it is critical for justice agencies (e.g., prisons, jails, community supervision programs) to provide clients with treatment services that address the physical and psychological consequences of substance use.

An aim of many substance use treatment programs is to provide clients with the support needed to achieve sustained recovery post-treatment (see McHugh et al., 2010 for a full review). Success in substance use treatment has been operationalized using reductions in substance use cravings (Davis et al., 2018), decreases in psychological distress (Erga et al., 2021), and the number of days abstinent (Daughters et al., 2018). Programs that primarily aim to eliminate undesirable symptoms related to substance use can be considered as functioning under a deficits-based paradigm (De Jong and Berg, 1998; Mott and Gysin, 2003). Alternatively, treatment programs working from a strength-based paradigm define recovery as a continual process including personal growth and sustained effort (e.g., McKenzie et al., 2016; Barnes-Lee, 2020; Best and Colman, 2020; Colman and Blomme, 2020; Fedock and Covington, 2022). This could include, for example, progress in substance use treatment involving improvements in resilience, self-confidence, and self-actualization (see Ezell et al., 2023 for a full review).

Meaning in life (MIL) plays an integral role in physical (Czekierda et al., 2017), psychological (Jin et al., 2016), and spiritual well-being (Kang et al., 2009; Aydın et al., 2020). Frankl (1984) theorized that persons without a clear sense of purpose find themselves in existential crisis and may trend towards maladaptive behaviors (e.g., substance use). Scholars have since discriminated the concepts “presence in meaning” and “search for meaning” (e.g., Steger et al., 2009), which relate differently to substance use. Presence of meaning in life describes the experience of knowing one’s life purpose and has been negatively associated with substance use (Thege et al., 2009; Csabonyi and Phillips, 2020). In contrast, search for meaning involves the active pursuit of one’s purpose and has been considered a risk factor for substance use (Ortíz and Flórez, 2016). In this way, helping clients clarify their purpose in life could lead to improved treatment outcomes. While controlling for age, baseline substance use, and depression, purpose in life was prospectively associated with less cocaine and alcohol use among people in a 30-day inpatient treatment program (Martin et al., 2011). Therefore, cultivating MIL presence may not only provide those involved with the legal system a more comprehensive, strength-based approach to recovery but also improve substance use treatment outcomes.

### 1.1 Treatment engagement

Success in substance use treatment is partially dependent upon providers’ capacity to engage clients early in treatment (see Simpson, 2004 for a full review). Treatment engagement can be assessed using measures of participation in counseling sessions, ratings of counselor rapport, or perceived peer support (e.g., Simpson and Joe, 2004; Pankow et al., 2012; Yang et al., 2018). Among legally involved persons, measures of treatment engagement have predicted improvements in motivation for changing substance use behavior (Simpson et al., 2012) and, post-treatment, less substance use and criminality (Joe et al., 2001). Peer support and counselor rapport, in particular, may be early indicators of engagement that provide a foundation for sustained engagement throughout treatment. For example, women in a substance use treatment (some legally involved) reported that positive peer support and a strong therapeutic alliance were facilitators of participation in group therapy sessions (Yang et al., 2018).

While treatment engagement has been correlated with improved motivation, less substance and alcohol use, and less criminality post-treatment (Harris et al., 2010; Miles-McLean et al., 2019), no study to our knowledge has assessed treatment engagement as a predictor of MIL. One study using a sample of detained youth found that females with higher physical and psychological well-being were more engaged in treatment (Van Damme et al., 2015), and related investigations have shown that people in substance use treatment do show positive changes in their quality of life (Maremmani et al., 2007; Pasareanu et al., 2015). Although predictors of improved quality of life were not assessed, conceptually it seems possible that these improvements in quality of life were dependent upon engagement in the therapeutic process. Thus, given the role that MIL presence has on substance use treatment outcomes (Thege et al., 2009; Ortíz and Flórez, 2016; Csabonyi and Phillips, 2020), it may be clinically meaningful to pinpoint novel ways of examining the relationship between treatment engagement and life meaning.

### 1.2 State of Surrender

State of Surrender (SoS), the willingness to accept what is to come without resistance, was originally described by James (1902) in Var*ieties of Religious Experiences*. James theorized that SoS is a psychological state that proceeds transformative-like experiences (James, 1902, pp. 189–216). Indeed, surrendering has been found to predict of mystical experiences for people participating in intensive meditation (Russ and Elliott, 2017). A more recent series of studies showed that a surrender state was correlated with indicators of psychological well-being (e.g., thriving, flourishing, happiness, life satisfaction; Sease et al., 2024). Additionally, this paper showed that SoS was closely related to, although statistically distinct from, psychological flexibility and mindfulness—two constructs that have been implicated as mechanisms of change in the substance use treatment (Li et al., 2017; Ii et al., 2019).

SoS has not been explored in the context of substance use, nor the transformative experiences involved in recovery. Substance use, however, has been described as an avoidant strategy for legally involved persons to cope with adverse experiences (e.g., Phillips and Lindsay, 2011; Binswanger et al., 2012; Johnson et al., 2013). This implies a surrender to adverse experiences and situations could promote positive change in substance use treatment. Here, building SoS would involve a shift in the clients’ repertoire where unwanted thoughts, feelings, and sensory experiences are no longer predictive of avoidance, rule-following, or other rigid behavior patterns related to substance use (Hayes and Brownstein, 1986). Instead, SoS would allow a person in substance use treatment to accept aversive experiences or situations and still choose to engage in behavior that aligns with their personally chosen goals or values. Therefore, SoS could be a psychological state that allows clients in treatment to elect for alternative, perhaps more functionally meaningful, behaviors that do not include substance use.

### 1.3 Current study

The purpose of the present study was to investigate whether SoS statistically mediated the relationship between measures of treatment engagement and self-reported MIL presence. We used a justice sample in substance use treatment to determine whether measures of treatment engagement were positively correlated with SoS scores. We expected that people reporting higher engagement in treatment, as indicated by assessments of treatment participation, counselor rapport, and peer support, would report higher levels of SoS. We also tested whether SoS was correlated with improvements in self-reported meaning in life presence while controlling for treatment engagement. Increases in treatment engagement were hypothesized to be correlated with increases in SoS, which in turn were expected to be positively correlated with MIL presence.

## 2 Method and materials

### 2.1 Participants

As illustrated in Table 1, the final sample included 60 males and 67 females (assigned sex at birth) who ranged in age from 21 to 66 (*M* = 37.14, *SD* = 9.97). Most participants were White (*n* = 65, 52.8%), non-Hispanic/Latino (*n* = 86, 69.9%), and had completed at least 12 years of schooling (*n* = 92, 74.8%). When asked, most people reported methamphetamine (*n* = 38, 30.9%) as the substance causing them the most difficulty in the past 12 months, followed by persons reporting more than one substance (*n* = 36, 29.3%), heroin (*n* = 16, 13.0%), and alcohol (*n* = 14, 11.4%). More than half (*n* = 67, 54.5%) of the sample had received substance use treatment in the past and about a third (*n* = 38, 30.9%) described their substance use as an extreme problem. The average length of time spent in treatment prior to completing the current study was 13 weeks (*SD* = 10.29), or just over 3 months.

**Table 1:** Demographics (*N* = 123).

	*n*	%
**Sex**		
Male	58	47.2%
Female	65	52.8%
**Hispanic**		
No	86	69.9%
Yes	37	30.1%
**Race**		
American Indian/Alaska Native	2	1.6%
Black/African American	27	22.0%
White	66	52.8%
Multiracial	8	6.5%
Other	14	11.4%
Not answered	7	5.7%
**Education**		
1–6	3	2.4%
7–9	12	9.8%
10–11	14	11.4%
12 or GED	58	47.2%
More than 12 years	34	27.6%
Not answered	2	1.6%
**Marital status**		
Single (never married)	73	59.3%
Married or living with partner	16	13.0%
Separated	8	6.5%
Divorced	21	17.1%
Widowed	2	1.6%
Not answered	3	2.4%
**Primary drug used**		
Alcohol	14	11.4%
Marijuana	5	4.1%
Heroin	16	13.0%
Cocaine	3	2.4%
Crack cocaine	6	4.9%
Prescription opioids	1	0.8%
Methamphetamine	38	30.9%
Hallucinogens	2	1.6%
More than one drug	36	29.3%
Other	2	1.6%
**Prior treatment experience**		
Yes	67	54.5%
No	54	43.9%
Not answered	2	1.6%
**How serious do you think your drug problems are?**		
Not at all	12	9.8%
Slightly	11	8.9%
Moderately	29	23.6%
Considerably	32	26.0%
Extremely	38	30.9%
Not answered	1	0.8%

Numbers represent totals and percentages.

### 2.2 Materials

#### 2.2.1 Sociodemographic information

A handful of items were used to collect background information about the people participating in this study. These items included questions asking about the participant’s age, assigned sex at birth, race, and education.

#### 2.2.2 State of surrender

State of Surrender was measured using an 8-item version of the SoS scale (Russ and Elliott, 2017). Using a 4-point Likert scale (1 = *Strongly Disagree*, 4 = *Strongly Agree*), participants were instructed to rate their agreement with each item as it reflected their psychological state during the past 2 weeks. The SoS scale measures one’s readiness to accept what is to come and has shown acceptable internal reliability (*α* = 0.86–0.89) and validity (Russ and Elliott, 2017; Russ et al., 2019a,b). In the current study, the 8-item measure had an acceptable internal consistency score (*α* = 0.91) and was scored by taking the sum of all items. Sample items for the SoS scale include, “I have stopped resisting and released control” and “I have now ceased straining.” The complete SoS assessment used in this study has been made publicly available at: https://tinyurl.com/y94hszpv.

#### 2.2.3 Treatment engagement

Treatment engagement was measured using the treatment participation, counselor rapport, and peer support measures of the TCU Engagement form (Institute of Behavioral Research, 2007). Using a 5-point Likert scale (1 = *Strongly Disagree*, 5 = *Strongly Agree*), participants were asked to rate their agreement or disagreement with each scale item. The treatment participation (i.e., You are willing to talk about your feelings during counseling), counselor rapport (i.e., You trust your counselor), and peer support (i.e., Other clients at this program care about you and your problems) scales have shown strong psychometric properties in legally involved samples (Joe et al., 2007; Simpson et al., 2012). Scale scores for treatment engagement measures were calculated by taking the sum of all items within each individual scale. The treatment participation (*α* = 0.91), counselor rapport (*α* = 0.96), and peer support (*α* = 0.84) scales all had acceptable internal reliability scores in the current study.

#### 2.2.4 Meaning in life

Meaning in Life was measured using the first 3 items on the presence of meaning subscale on the Meaning in Life Questionnaire (Steger et al., 2006). Due to an experimenter error, the Meaning in Life Questionnaire was presented on a 3-point Likert scale (1 = *Strongly Disagree*, 2 = *Neither Agree not Disagree*, 3 = *Strongly Agree*). Participants were instructed to rate how much they agreed or disagreed with each item and the 3-item scale had an internal reliability score of 0.82. Example items include, “I understand my life’s meaning,” and, “I have a good sense of what makes my life meaningful.” Meaning in life scale scores were calculated by taking the sum of all items, with a higher score representative of more MIL.

### 2.3 Procedure

The first author contacted the correctional facility and requested permission to collect de-identified data from clients participating in substance use treatment. Flyers were posted around the facility and potential participants with an interest in the study were instructed to notify a staff member who kept a running list of everyone who wanted to participate. On the day of data collection, correctional staff helped coordinate meetings between the research staff and study participants, so that data collection could be completed in small groups (i.e., 5–8 participants at a time). Study sessions lasted around 35 min and consisted of participants being asked to complete a paper survey created with SnapShot - a commercial software that converts data on paper surveys to an electronic data file. The first author was available throughout study sessions to answer questions about the study, survey items, and/or read the study questions to participants who could not read or had a visual impairment. The treatment program provided to clients at this facility is a modified cognitive behavioral intervention that uses motivational interviewing, cognitive reframing, and behavioral modification techniques to promote positive changes in substance use behavior. Clients receive an average of 20 h of programming a week, which includes substance use programming and additional classes intended to supplement substance use treatment (e.g., anger management and skills training). Participation in this study did not affect contact extent or type of programming. All participants in this study provided an informed consent prior to the start of the study and were debriefed following the completion of the study.

### 2.4 Analytic plan

Analyses were conducted using SPSS and the PROCESS 4.0 macro (Model 4; Hayes, 2018). Demographics (see Table 1) and descriptive statistics for all variables of interest were calculated and correlation analysis explored the relationships among all variables of interest (Table 2). Next, three separate models assessed the mediational effect of SoS on the relationships between (1) peer support, (2) treatment participation, and (3) counselor rapport with meaning in life. All results of mediation analyses are interpreted while controlling for days in the facility. A Monte Carlo sensitivity analysis (Schoemann et al., 2017) using a sample size of 127 showed that our study had between 92 and 95% power to detect an indirect effect when predicting MIL presence.

**Table 2:** Correlational analyses.

Variable	Mean (*SD*)	1	2	3	4	5
1. Peer Support	17.54 (4.14)					
2. Treatment Participation	49.54 (7.77)	0.44**				
3. Counselor Rapport	48.02 (9.70)	0.40**	0.57**			
4. State of Surrender	23.81 (4.77)	0.54**	0.52**	0.46**		
5. Meaning in Life	22.96 (4.06)	0.35**	0.35**	0.34*	0.42**	
6. Weeks in Treatment	13.00 (10.29)	−0.08	−0.13	−0.18*	−0.28**	−0.10

*p < 0.05; **p < 0.001.

## 3 Results

**Figure 1:**
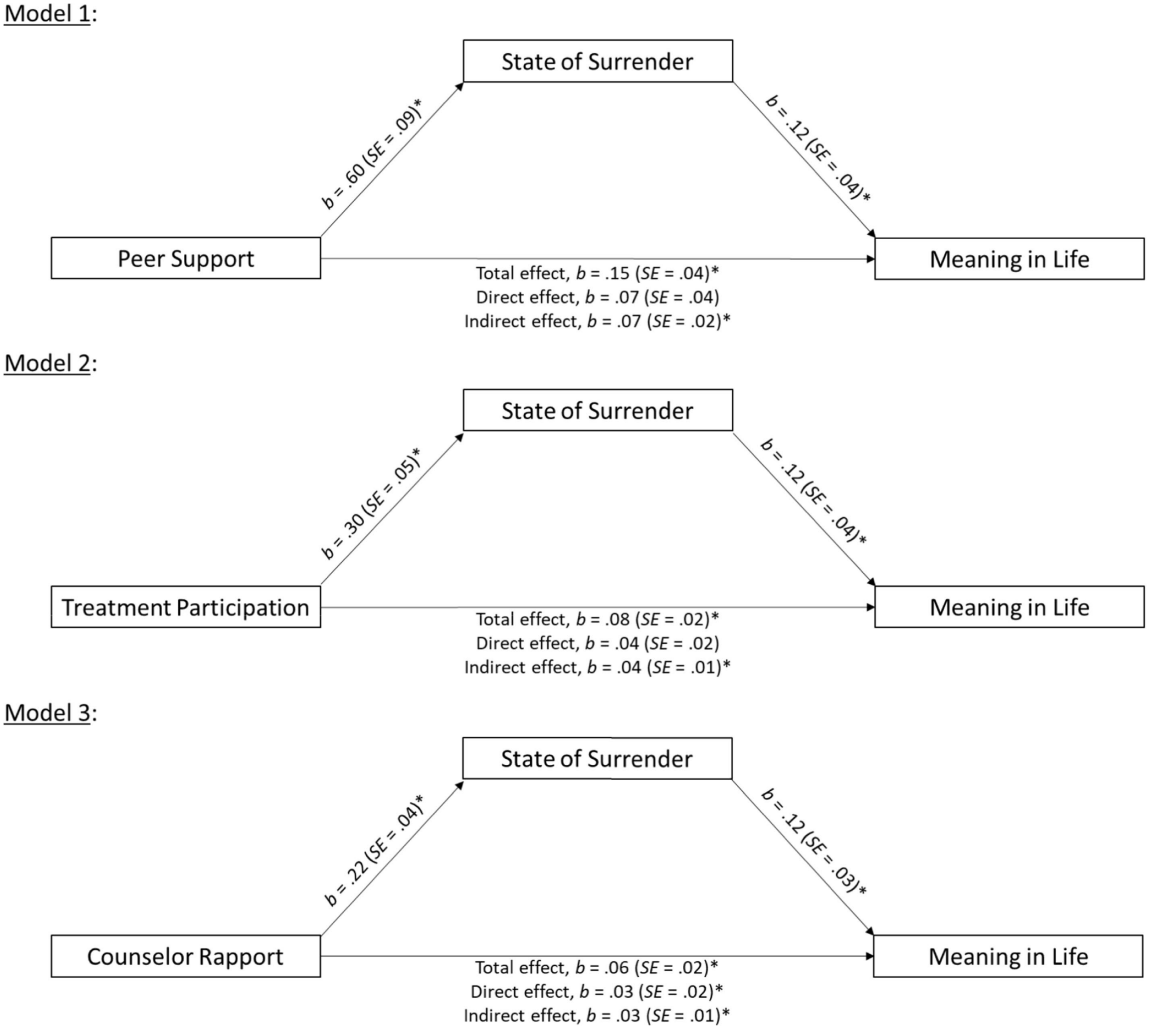
Path models for the relationship between peer support (Model 1), treatment participation (Model 2), and counselor support (Model 3) on MIL as a function of SoS. All results are controlling for number of days in the treatment facility. Unstandardized coefficients (and standard errors) are depicted in all models. *p < 0.05.

Following MacKinnon and Luecken (2008), three mediation analyses were performed to examine whether SoS influenced the relationship between peer support (Model 1), treatment participation (Model 2), and counselor rapport (Model 3) on MIL. Number of days in the treatment facility was included as a covariate in all analyses. Inferential statistics are reported in Table 3, while graphical depictions of the mediational models are in Figure 1. The a-paths for all models were statistically significant, with peer support, treatment participation, and counselor rapport all predicting greater SoS. Examination of the b-paths revealed there was also a positive relationship between SoS and MIL while controlling for peer support (Model 1), treatment participation (Model 2), and counselor rapport (Model 3). Finally, utilizing 5,000 bootstrap resamples, the 95% confidence intervals for the indirect effects were significant for all models: peer support [0.030, 0.120]. treatment participation [0.015, 0.062], and counselor rapport [0.011, 0.045]. Taken together, these findings suggest that peer support, treatment participation, and counselor rapport are all associated with a greater SoS, which in turn is related to greater MIL.

**Table 3:** Results of SoS mediating the relationship between peer support (Model 1), treatment participation (Model 2), and counselor rapport (Model 3) on MIL while controlling for number of days in the treatment facility.

	***b***	***SE***	***t***	***p***-value	***95% CI***
**Model 1**					
Peer support on SoS (*a* path)	0.60	0.09	6.96	< 0.001	0.430, 0.773
SoS on MIL (*b* path)	0.12	0.04	3.27	0.001	0.047, 0.192
Peer support on MIL (*c* path)	0.15	0.04	4.04	< 0.001	0.074, 0.217
Peer support on MIL via SoS (*c*’path)	0.07	0.04	1.79	0.076	−0.008, 0.155
**Model 2**					
Treatment participation on SoS (*a* path)	0.30	0.05	6.40	< 0.001	0.207, 0.393
Treatment participation on MIL (*b* path)	0.12	0.04	3.39	0.001	0.050, 0.192
Treatment participation on MIL (*c* path)	0.08	0.02	3.98	< 0.001	0.038, 0.114
Treatment participation on MIL via SoS (*c*’path)	0.04	0.02	1.87	0.064	−0.002, 0.082
**Model 3**					
Counselor rapport on SoS (*a* path)	0.22	0.04	5.33	< 0.001	0.137, 0.299
Counselor rapport on MIL (*b* path)	0.12	0.03	3.63	< 0.001	0.057, 0.192
Counselor rapport on MIL (*c* path)	0.06	0.02	3.85	< 0.001	0.030, 0.094
Counselor rapport on MIL via SoS (*c*’path)	0.03	0.02	2.04	0.044	0.001, 0.068

## 4 Discussion

People with a history of involvement in the legal system are at heightened risk of developing substance use disorders (Bronson et al., 2017; Chamberlain and Boggess, 2019; Zgoba et al., 2020), and substance use in legal samples contributes to worsened physical and psychological well-being (Keaney et al., 2011; Lai et al., 2015; McKetin et al., 2019; Colledge et al., 2020). The legal system is therefore in an optimal position to provide people in jail or prison with treatment services for substance use. Strength-based paradigms of recovery emphasize the importance of developing resilience, self-confidence, and self-actualization (e.g., Ezell et al., 2023). Meaning in Life presence, for example, has been documented as a protective factor against substance use (Thege et al., 2009; Ortíz and Flórez, 2016; Csabonyi and Phillips, 2020) and could be one way of assessing recovery from a strength-based perspective. As such, the current study investigated correlates (e.g., treatment participation, counselor rapport, social support) of presence in meaning among legally involved persons participating in residential substance use treatment. We also considered SoS—the willingness to accept what is to come, good or bad—as a statistical mediator of the relationship between treatment engagement and MIL.

We hypothesized that measures of treatment participation would be positively associated with SoS scores, and that SoS scores, in turn, would be associated with improved MIL presence. Measures of treatment engagement, including treatment participation, counselor rapport, and peer support, were positively correlated with SoS. Measures of treatment engagement explained about a fifth (*R*^2^s ≥ 21.6%) of the observed variance in SoS scores, suggesting that engaging clients early in treatment may be one way to facilitate improvements in SoS. Treatment engagement has been associated with improved treatment outcomes in legal samples (Harris et al., 2010; Miles-McLean et al., 2019), and the present study would suggest that SoS could be impacting these relationships. In support, SoS was associated with greater presence in meaning, while controlling for the effects of treatment engagement. When treatment engagement fosters MIL, it may do so by providing the context for clients to surrender. More specifically, early indicators of engagement in individual or group therapy sessions may serve as contextual cues that clients can be comfortable surrendering in treatment without fear of negative consequences (e.g., therapist judgment, negative peer evaluations).

Providers may be able to use SoS, along with other known processes of change in substance use treatment, to (1) attenuate undesirable substance use symptoms, and (2) promote clients’ psychological well-being. Incorporated alongside existing empirically-supported treatment protocols (e.g., motivational interviewing, therapeutic community programs, group therapy), implementing practices that evoke SoS would be consistent with a more comprehensive, strength-based treatment approach. The legal system has historically considered the role of confinement as punitive (see Phelps, 2011), making the implementation of strength-based treatment programs a warranted and timely endeavor. Strength-based treatment programs incorporated in legal settings have yielded favorable outcomes (e.g., Hunter et al., 2015; Best et al., 2018; Hall et al., 2018). The effectiveness of strength-based models of care in legal settings provides indirect support for the acceptability and feasibility of interventions that target SoS.

In practice, SoS may offer providers a manipulable process that improves the well-being of clients involved with the legal system. Surrendering to the challenges that recovery presents may allow clients to move past difficult experiences and reorient themselves towards what they find meaningful in life. This process may be closely related to how acceptance- and mindfulness-based exercises facilitate recovery for persons with substance-related difficulties (see Li et al., 2017; Chen, 2022). Moreover, given the conceptual overlap, future studies may consider using acceptance- and mindfulness-based techniques to elicit a surrender state, and move clients toward their treatment goals. Data from our lab has shown that a surrender state can be precipitated using a brief 10-min mindfulness exercise (Sease et al., 2024; Study 3), indicating that similar exercises may be useful for evoking SoS in clinical settings.

### 4.1 Limitations and future directions

This study administered a single-session self-report survey to legally involved persons in substance use treatment to explore the relationships among SoS, treatment engagement, and MIL. This cross-sectional design eliminates the possibility of inferring causation from the proposed mediation models and establishing a temporal relationship among the variables of interest (see O’Laughlin et al., 2018 for a more detailed discussion on this topic). Forthcoming studies will need to replicate these findings and demonstrate that SoS is associated with improvements in substance-related behaviors prospectively. Such studies may pinpoint SoS’s role in the process of recovery by assessing progress in substance use treatment both in terms of reductions in substance use behavior and improvements in psychological well-being. It remains undetermined as to whether increases in SoS are important for the alleviation of substance use symptoms or strictly related to improvements in psychosocial well-being. Additionally, this line of research would largely benefit from introducing an acceptance-based intervention to see whether precipitating SoS (as compared to a control condition) would increase well-being, treatment adherence, and long-term outcomes in people recovering from substance use.

Due to time limitations, we were unable to administer Steger et al.’s (2006) entire 10-item MIL questionnaire. This is important as the scale assesses both presence (i.e., the belief that someone has experienced meaning in one’s present life) and search for meaning (i.e., motivationally seeking meaning in one’s life). Research has found that people scoring high on a search for meaning are more likely to report lower emotional and psychological well-being, including higher levels of anxiety, depression, stress, anger, hostility, and fear (Steger et al., 2006, 2008). It could be that persons using substances are particularly vulnerable to a greater search for meaning, and consequently, lower health and treatment outcomes given their tendency to have poorer relationships, lower self-acceptance, and an increased openness in experimenting with drugs and alcohol (e.g., Csabonyi and Phillips, 2020). Regarding the current results, an inability to surrender and engage in residential treatment may be associated with greater meaning search, reduced health, and increased substance use and recidivistic outcomes.

The present study also suffers from a limited sample size, raising justifiable concerns about the stability and generalizability of this study’s findings. Therefore, it is imperative that this study is replicated and extended using a larger sample that is more representative of people with substance-related difficulties. Perhaps the largest limitation of the present study is the limited empirical evidence demonstrating that SoS can be manipulated in experimental settings, and SoS’s potential utility in clinical populations. More research is needed to determine both the short- and long-term effects SoS may have for people in substance use treatment. For example, SUDs are typically chronic conditions that require long-term interventions to see durable change. If SoS is the mechanism by which clients in residential treatment can adhere to therapy and improve health and well-being, then a surrender state may need to be practiced daily over time to prevent relapse and reduce persons’ substance use. Additional work is thus needed to test the effectiveness of both brief versus long-term SoS interventions on sustained substance use treatment and recovery.

## 5 Conclusion

The current study is the first to explore how SoS may aid with progress in substance use treatment for people who are legally involved. This is important as people with a history of involvement with the legal system are at heightened risk of developing SUDs (Bronson et al., 2017; Chamberlain and Boggess, 2019; Zgoba et al., 2020), and problems with substance use can increase a person’s chance of returning to criminal activity post-release (Zgoba et al., 2020). As hypothesized, SoS statistically mediated the relationship between measures of treatment engagement, including treatment participation, counselor rapport, peer support, and MIL. Treatment engagement was associated with more SoS, which in turn was associated with greater MIL. These results suggest that SoS may be a targetable process in substance use treatment engagement that aids in recovery by orienting clients toward what they find meaningful in life. Clinical providers may consider cultivating SoS in substance use treatment to improve clients’ life meaning presence.

## Data availability statement

The raw data supporting the conclusions of this article will be made available by the authors, without undue reservation.

## Ethics statement

The studies involving humans were approved by The Institutional Review Board at Texas Christian University. The studies were conducted in accordance with the local legislation and institutional requirements. Written informed consent for participation in this study was provided by the participants' legal guardians/next of kin.

## Author contributions

TS: Conceptualization, Data curation, Investigation, Writing – original draft, Writing – review & editing. CC: Conceptualization, Data curation, Investigation, Supervision, Writing – original draft, Writing – review & editing. AW: Formal analysis, Software, Visualization, Writing – original draft, Writing – review & editing. ES: Writing – original draft, Writing – review & editing. KK: Resources, Supervision, Writing – review & editing.
